# A Tri-Band Omnidirectional Shark-Fin Antenna for Vehicle Applications: Design and Analysis

**DOI:** 10.3390/mi17070871

**Published:** 2026-07-22

**Authors:** Chong-Zhi Han, Zhanhong Qiu, Jun Xiao, Pengyu Zhang, Wei He, Ziji Zhang, Lu Liu

**Affiliations:** 1 School of Ocean Information Engineering, Jimei University, Xiamen 361021, China202412854042@jmu.edu.cn (Z.Q.); xiaojun@jmu.edu.cn (J.X.);; 2 Nanjing Zhongpu Weida Electronic Technology Co., Ltd., Nanjing 210000, China; pyzhang1987@hotmail.com; 3 School of Safety and Management Engineering, Hunan Institute of Technology, Hengyang 421002, China; hewei21801@163.com

**Keywords:** shark-fin antenna, vehicular communications, printed dipoles, wideband antenna

## Abstract

In this paper, a tri-band omnidirectional shark-fin antenna for vehicular communications is proposed, which can cover three operating bands: Ultra High Frequency (UHF, 400–470 MHz), LTE Band 5 (824.2–879.2 MHz), and LTE-1800 (1765–1880 MHz). The antenna integrates a central UHF monopole and a pair of symmetric printed radiating elements within a compact shark-fin radome. The printed elements excite independent resonant modes in each band by using T-shaped and I-shaped radiating branches; broadband matching and balanced excitation are realized through a tapered impedance transformation network; and a dual-band array configuration is adopted to improve gain and stabilize radiation patterns. The monopole and printed elements form a collaborative array in a limited space, achieving structural miniaturization while obtaining good isolation and omnidirectional radiation characteristics. Results show that the peak gain of the antenna is 2.3 dBi in the UHF band and 7.2 dBi in the LTE-1800 band, and the isolation is better than 10 dB. The measured results are consistent with simulations, verifying the feasibility of the proposed design for application in high-performance vehicular communication systems.

## 1. Introduction

With the full-scale implementation of intelligent connected vehicles, vehicle-to-everything (V2X) interaction, 5G-Advanced and integrated sensing, and communication (ISAC) technologies impose stringent comprehensive performance constraints on roof-integrated shark-fin antennas [1,2,3,4,5,6]. Restricted by vehicle aerodynamic styling, all radiating assemblies must be packaged inside compact shark-fin radomes. The antenna design is required to satisfy four core specifications simultaneously: multi-band compatibility, stable omnidirectional radiation on the horizontal plane, high realized gain, and sufficient port isolation. Dense arrangement of multiple radiators within a confined cavity will induce intense near-field electromagnetic coupling, which distorts azimuth radiation patterns, reduces radiation efficiency, and degrades communication reliability. Accordingly, balancing multi-band coverage, uniform horizontal radiation, high gain, and low inter-element mutual coupling under limited packaging space has become a core research challenge in the field of vehicular shark-fin antennas, attracting extensive research attention from scholars worldwide [7,8,9,10,11,12,13].

To achieve multi-band response of vehicular antennas, various structural design paradigms have been proposed in existing references [14,15,16,17,18,19,20], among which the mainstream implementation approaches fall into four categories: printed multi-resonant branches, sleeve-loaded monopoles, parasitic patch coupling, and defected ground structure (DGS) impedance tuning. In terms of printed radiating architectures, Ref. [14] adopts segmented T-shaped and I-shaped branches to excite independent resonant modes, covering dual frequency bands of 617–960 MHz and 1710–6000 MHz. Nevertheless, this design only adapts to mid-to-high long-term evolution (LTE) bands without ultra high-frequency (UHF) or low-frequency coverage. Ref. [15] presents an L-sleeve L-monopole shark-fin antenna that broadens the operating bandwidth via electromagnetic coupling between the main radiator and the sleeve, supporting both LTE and 5–6 GHz vehicle-to-everything frequency bands, while its lowest cut-off frequency remains higher than 698 MHz. The classical polygonal monopoles and planar PIFAs reported in Refs. [16,17] generate multiple resonant points by bending radiating branches to realize dual LTE bands ranging from 824 to 2175 MHz, which still fail to meet the communication requirements of the special 400–470 MHz vehicular frequency band. By extending current paths through multi-stage elongated branches, the antenna in Ref. [18] achieves ultra-wideband coverage from 617 MHz to 5 GHz. Apart from all-printed radiators, metasurface loading has been verified as effective for impedance bandwidth expansion [19,20], yet additional metasurface layers increase vertical thickness and cannot be embedded into standard shark-fin radomes. In summary, most multi-band shark-fin antennas are designed for cellular communication bands above 700 MHz, lacking compatible schemes for the 400–470 MHz UHF private network. Moreover, multi-layer stacking and external matching networks will enlarge the overall size and aggravate inter-element coupling. To fill this research gap, this paper proposes a composite radiating architecture consisting of a central vertical UHF monopole and a pair of symmetric double-sided printed antennas. The central metallic monopole generates fundamental resonance for the 400–470 MHz band. Each dielectric substrate is etched with T-shaped low-frequency branches and I-shaped high-frequency branches to independently excite resonant modes of LTE Band 5 (824.2–879.2 MHz) and LTE-1800 (1765–1880 MHz). A tapered microstrip balun integrated on the back of the substrate realizes passive wideband impedance compensation. Synchronous matching of three discrete frequency bands can be realized without extra matching circuits, enabling tri-band integration within a limited shark-fin cavity.

For the omnidirectional design of vehicular antennas, a wealth of mature omnidirectional design strategies have been summarized in existing antenna research [8,14,18,21,22,23]. The first type of scheme adopts standalone vertical monopoles to achieve inherent omnidirectional performance via ground mirror effects. Nevertheless, as stated in Refs. [8,18], metal vehicle roofs and plastic radomes induce intense electromagnetic reflection, leading to prominent gain nulls at high frequencies with the maximum azimuth fluctuation exceeding 3.0 dB. The second mainstream strategy employs two symmetric radiators for superimposed and complementary field superposition. Refs. [21,22] arrange a pair of Vivaldi or PIFA elements in mirror layouts to counteract azimuth nulls, restricting H-plane fluctuation within 1.8–1.9 dB. However, independent metal shielding partitions between radiators occupy substantial internal space and leave limited room for multi-band branches. The third category relies on circular multi-element arrays to synthesize quasi-omnidirectional radiation [14,24]. Mutual blockage among array elements deteriorates radiation uniformity, and the peak gain fluctuation across the full bandwidth reaches up to 3.7 dB. Although transmissive metasurfaces can theoretically reshape azimuth beams [24], their excessive structural thickness renders them unsuitable for miniaturized shark-fin applications. All the above architectures suffer from inherent drawbacks: additional shielding components sacrifice packaging volume, high-frequency pattern distortion is hard to avoid, and none can maintain uniform radiation performance at low UHF frequencies. Different from conventional symmetric array and single-monopole paradigms, this paper innovatively proposes a collaborative radiation scheme combining a central vertical monopole and two symmetric printed antennas. The central metallic column generates uniform horizontal radiation at 400–470 MHz, while the two side-printed radiators produce leftward and rightward radiation at 824.2–879.2 MHz and 1765–1880 MHz respectively. Complementary superposition of radiated fields realizes omnidirectional coverage for the two LTE bands. Stable omnidirectional radiation can be achieved without any auxiliary shielding structures in the proposed design.

Radiation gain enhancement serves as a universal optimization objective for all types of wireless antennas. Four mainstream gain-improving approaches are widely adopted, including in-phase array superposition, parasitic director patch loading, double-sided printing for equivalent electrical aperture expansion, and passive compensation based on tapered feedlines. The four-element multiple-input multiple-output (MIMO) array proposed in Ref. [18] boosts gain through the superposition of radiated fields from multiple elements, yet its overall dimensions exceed the installation limits of standard shark-fin radomes. Although parasitic metal directors can concentrate beams and enhance directivity, they increase the antenna profile height and damage the vehicle’s aerodynamic appearance. Double-sided coplanar PCB printing stands out as one of the optimal gain optimization methods for miniaturized antennas. The vehicular printed antennas in Refs. [15,21] both adopt dual-layer radiating structures to enlarge the effective radiation area. Meanwhile, tapered microstrip feedlines smooth impedance variations and reduce feed loss, thereby indirectly improving radiation efficiency [24,25]. In contrast, most existing shark-fin vehicular antennas utilize single-layer narrow radiating branches with small equivalent electrical apertures, resulting in peak gain lower than 6 dBi across the whole operating band and prominent gain discrepancies between low and high frequencies. Different from conventional single-frequency arrays with narrow bandwidth, the proposed antenna forms an array structure to elevate in-band gain. T-shaped and I-shaped branches are printed on both the upper and lower surfaces of the dielectric substrate simultaneously to form a compact dual-band coplanar array, which achieves gain enhancement across the entire operating bandwidth while reducing the antenna footprint.

Dense arrangement of multiple radiators within the compact shark-fin cavity leads to severe spatial mutual coupling, which reduces MIMO diversity throughput. Four mainstream decoupling strategies have been developed for existing vehicular MIMO antennas, each with inherent drawbacks. First, decoupling via defected ground structure (DGS): Refs. [14,23] etch irregular slots on the ground plane to suppress surface wave propagation, which only achieves port isolation of 10–13 dB and delivers poor decoupling performance at low frequencies. Second, orthogonal polarization design: Ref. [23] adopts dual-polarized radiators to mitigate coupling through electric field orthogonality, yet polarization purity varies across different frequency bands and mutual coupling deteriorates at high frequencies. Third, standalone metal shielding partitions: Ref. [18] installs metal baffles between antenna elements to boost isolation above 14 dB, but such shielding structures occupy internal space reserved for multi-band radiating branches. Fourth, external LC matching-decoupling networks: Ref. [26] connects passive decoupling circuits at feeding ports, which introduces extra insertion loss and degrades overall radiation efficiency. Additionally, metasurface-assisted decoupling schemes [19,27] increase substrate thickness and manufacturing costs. All the decoupling methods rely on extra auxiliary components, making it difficult to realize tri-band integration inside a limited radome. Unlike various additional decoupling configurations, the proposed antenna achieves passive wideband high isolation by virtue of its inherent spatial layout. The continuous central metallic monopole naturally acts as an electromagnetic barrier to block line-of-sight near-field coupling between the left and right printed dipoles. Meanwhile, the central radiator radiates vertical polarization while the tilted printed elements on both sides mainly generate horizontally polarized waves, forming a dual decoupling mechanism based on intrinsic polarization diversity. The port isolation exceeds 20 dB across all operating bands, and the envelope correlation coefficient remains below 0.2 throughout the full bandwidth. The proposed design satisfies the diversity requirements of vehicular MIMO systems without any slots, shielding plates or external circuits.

In view of the characteristics and existing limitations of vehicular antennas, this paper proposes a compact tri-band omnidirectional MIMO shark-fin antenna compatible with UHF and multiple LTE standards. The antenna adopts a composite radiating architecture consisting of a central vertical monopole and symmetric tilted printed dipoles. Independent resonant matching for three discrete frequency bands is realized via composite resonant structures, and complementary horizontal electric fields are utilized to mitigate azimuth gain dips. Passive high isolation is achieved through the synergistic effect of metallic shielding and polarization diversity. Simulated results are in good agreement with anechoic chamber measurements. The proposed antenna simultaneously achieves miniaturization, stable omnidirectional coverage, high gain and low mutual coupling, exhibiting comprehensive performance advantages for multi-scenario intelligent connected vehicle communication systems.

The remainder of the paper is organized as follows. Section 2 presents the proposed antenna design. Section 3 covers simulations and measurements. Section 4 provides a discussion.

## 2. Design of the Combination Antenna

**Figure 1:**
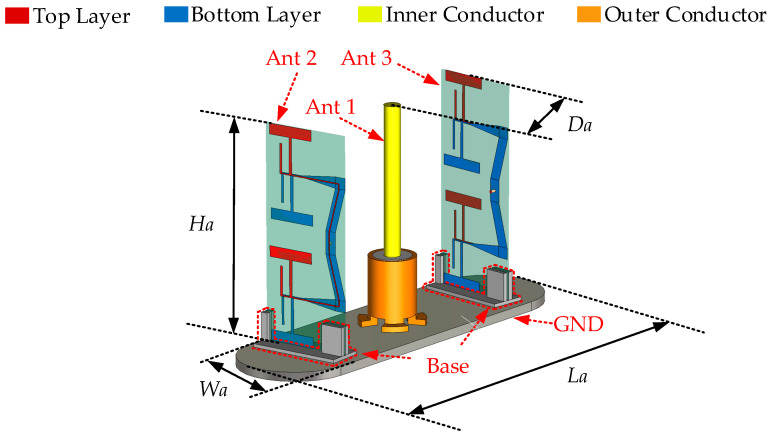
Overall configuration and dimension of the proposed shark-fin antenna.

To meet the requirements of modern vehicular mobile communications, the antenna should be capable of operating over multiple frequency bands. In this paper, a shark-fin vehicular antenna is proposed, which covers the UHF (400–470 MHz), LTE Band 5 (824.2–879.2 MHz), and LTE-1800 (1765–1880 MHz) bands. The overall dimensions of the proposed shark-fin antenna are *L*_a_ × *W*_a_ × *H*_a_. The complete configuration and dimensions are shown in Figure 1 and Table 1. The antenna mainly consists of a base, two identical printed antennas, and a monopole. The printed antennas are implemented on TU865 substrates (ε_r_ = 4.3, tanδ = 0.01). The antenna base is used to mechanically support the three antenna elements. The two printed antennas are mounted on both sides of the base with a non-parallel arrangement, tilted by ±5°, so that they can provide complementary radiation to achieve omnidirectional high gain while covering the 824.2–879.2 MHz and 1765–1880 MHz bands. The monopole, located at the center of the base, is employed to cover the 400–470 MHz band. The commercial software CST Studio Suite 2022 is used to optimize the dimensions of the entire antenna.

### 2.1. Monopole

**Figure 2:**
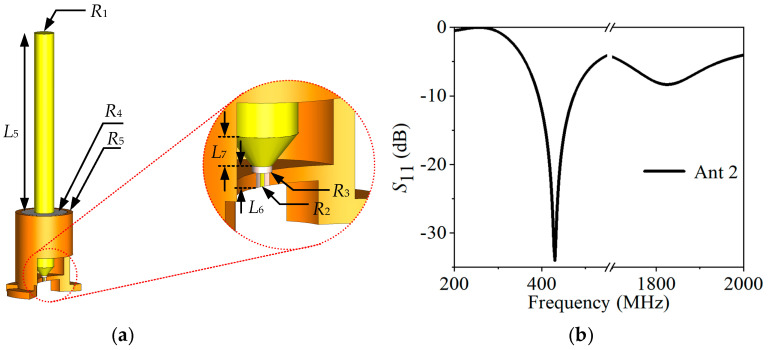
Dimensions and *S*-parameters of the UHF monopole antenna: (**a**) Configuration of the UHF monopole antenna; (**b**) *S*_11_ of the UHF monopole antenna.

The detailed configuration of the UHF monopole antenna is shown in Figure 2. The radiator is implemented as a metallic cylindrical rod with length *L*_5_ and radius *R*_1_, which is vertically mounted at the center of the metallic base and operates as a quarter-wavelength monopole in the 400–470 MHz band. The gray section in the middle of the radiator together with the orange part at the bottom forms the antenna base, ensuring the continuity and stability of the RF ground. In the transition region between the radiator and the base, a flared metallic section with inner radius *R*_4_ and outer radius *R*_5_ is adopted, and height *L*_7_ is introduced to provide a smooth impedance transition between the coaxial feed and the monopole, while effectively increasing the electrical top loading at the bottom of the monopole and thereby extending the operating bandwidth. The *S*-parameters of the UHF monopole antenna are shown in Figure 2. The antenna achieves a reflection coefficient |*S*_11_| < –10 dB across the 400–470 MHz band, yielding a fractional bandwidth of 16.3%.

Below the flared section, a short metallic post with height *L*_6_ is arranged. This post forms a coaxial feeding structure together with the metal cavity bounded by inner radius *R*_2_ and outer radius *R*_3_. This coaxial cavity confines the current distribution in the feeding region, suppressing spurious radiation from the feed line and reducing coupling to the surrounding antenna elements. The composite structure not only provides good impedance matching and stable omnidirectional radiation characteristics, but also satisfies the mechanical strength and reliability requirements for vehicular applications.

**Figure 3:**
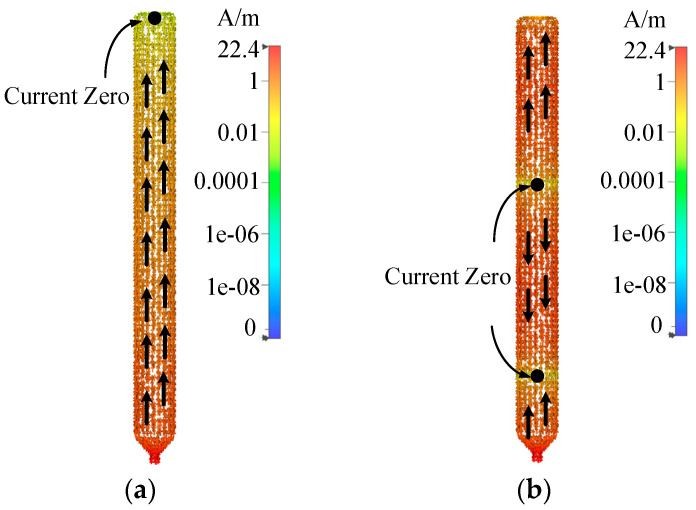
Surface-current distribution on the antenna conductor: (**a**) surface-current distribution on the monopole at 430 MHz; (**b**) surface-current distribution on the monopole at 1820 MHz.

As illustrated in Figure 2b, the antenna exhibits two resonant frequencies at 430 MHz and 1820 MHz, respectively. To elucidate the underlying mechanisms, the surface current distributions were investigated. As shown in Figure 3a, the current magnitude at the top of the monopole is null at 430 MHz, whereas Figure 3b reveals two current nulls along the structure at 1820 MHz. These observations confirm that the resonance at 430 MHz is attributed to the first-order mode, while the resonance at 1820 MHz corresponds to the third-order mode.

### 2.2. Printed Antennas

**Figure 4:**
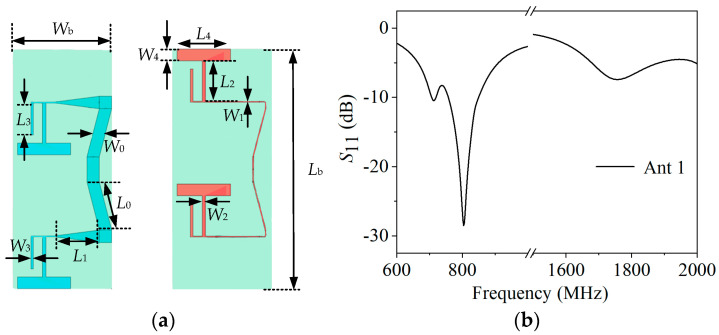
Dimensions and *S*-parameters of the printed antenna: (**a**) geometry and key dimensions; (**b**) simulated *S*-parameters.

The geometry of the printed antenna is shown in Figure 4a. The antenna adopts a two-layer metallic configuration on a dielectric substrate. The upper layer integrates the feed line and radiating elements, while the lower layer acts as the reference ground for the feeding and impedance transformation network. A dielectric substrate (TU865, ε_r_ = 4.3, tanδ = 0.01) is sandwiched between the two metal layers, forming a typical microstrip radiating element. The upper conductor is composed of a meandered main feed line and two branched radiators, while the lower layer incorporates a microstrip balun/impedance transformer together with the mirror image of the dual-branch monopole. The T-shaped red branch on the upper layer and the T-shaped blue structure on the lower layer are spatially combined to form an equivalent dipole whose electrical length is designed to be close to one half of the wavelength. The S-parameters of the proposed antenna are shown in Figure 4b. The main antenna finally covers two operating bands, namely LTE Band 5 (824.2–879.2 MHz) and LTE-1800 (1765–1880 MHz).

**Figure 5:**
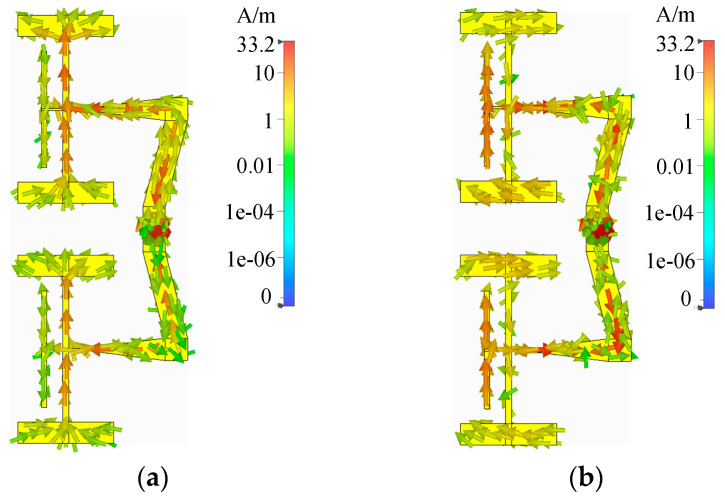
Surface current distributions of the printed antenna: (**a**) surface current distribution at 830 MHz; (**b**) surface current distribution at 1800 MHz.

From the surface current distribution of Figure 5, it can be observed that the dominant current path at this band corresponds to the fundamental mode with an effective electrical length of approximately 0.52*λ*, which generates and controls the low-frequency resonance covering 824.2–879.2 MHz. The upper I-shaped red branch is relatively shorter; its surface current distribution also indicates excitation of the fundamental mode, with a dominant current path of about 0.494*λ*. This branch is mainly responsible for forming the high-frequency resonance in the 1765–1880 MHz band, thereby realizing radiation in the LTE-1800 band. By printing the T-shaped and I-shaped radiating elements on the top microstrip layer, a dual-branch monopole is realized, and the combination of the top microstrip line with the tapered structure on the bottom layer provides improved input impedance matching for the overall antenna. The main antenna finally covers two operating bands, namely LTE Band 5 (824.2–879.2 MHz) and LTE-1800 (1765–1880 MHz).

To further verify the operating principle and validity of the design, the surface current distributions at 830 MHz and 1800 MHz are presented in Figure 5. At 830 MHz, the surface current is mainly concentrated along the main feed line, the upper T-shaped branch, and the corresponding T-shaped branch on the lower layer, forming a low-frequency resonant path whose electrical length is close to one half wavelength, whereas only weak current appears on the I-shaped branch. This confirms that the low-frequency resonance is predominantly contributed by the T-shaped branches, while the I-shaped branch plays only a minor tuning role in this band. In contrast, at 1800 MHz, the strong-current region clearly shifts to the I-shaped branch and the nearby short current paths; large currents flow on both sides of the vertical arm of the I-shaped branch, indicating a high-frequency resonant mode, whereas the T-shaped branches and the lower structures carry much weaker current and mainly act as parasitic loads. This demonstrates that radiation in the 1765–1880 MHz high-frequency band is dominated by the I-shaped branch, and the low-frequency T-shaped branches have little influence on this resonance. The two current distributions clearly verify that the T-shaped branch is responsible for the low-frequency resonance in the LTE Band 5 band, whereas the I-shaped branch generates the high-frequency resonance in the LTE-1800 band. Although both branches share the same feed point, their resonant mechanisms remain relatively independent, which is beneficial for dual-band tuning and good impedance matching. It should be noted that, owing to the mutual coupling between the branches, the actual electrical lengths of the resonant paths deviate slightly from the ideal half-wavelength values.

### 2.3. Feeding Network

**Figure 6:**
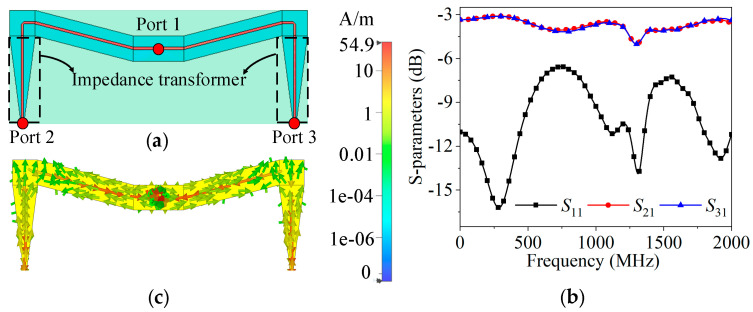
Impedance transformer, *S*-parameters, and surface current distribution of the proposed antenna: (**a**) geometry of the impedance transformer; (**b**) *S*-parameters; (**c**) surface-current distribution on the impedance transformer.

As illustrated in Figure 6a, a tapered microstrip impedance transformer is introduced at the bottom layer of the antenna to achieve broadband matching between the 50 Ω coaxial feed and the dual-branch printed monopole. The microstrip line width is gradually varied along its path and folded symmetrically at both ends, forming a pair of mirrored tapering arms. This geometry produces a continuous variation in characteristic impedance, enabling a smooth transition of the input impedance and, consequently, a significant reduction in the reflection coefficient within the two operating bands, while simultaneously extending the impedance bandwidth. The measured *S*-parameters of the transformer are plotted in Figure 6b. |*S*_21_| and |*S*_12_| remain close to −3 dB, whereas |*S*_11_| is maintained below −10 dB across the band of interest, indicating a stable and well-matched transition. Moreover, the left–right symmetric layout intrinsically behaves as a balun, impressing equal-amplitude, anti-phase currents on the two branches. This suppresses the common-mode current on the feed line, mitigates spurious radiation, and stabilizes the radiation pattern of the antenna. The surface-current distribution of the transformer is depicted in Figure 6c. The current density progressively increases as the line width narrows, validating the design prediction. The guided power is efficiently converted and smoothly coupled into free space, leading to an improved radiation performance.

### 2.4. Isolation Analysis Among Antennas

**Figure 7:**
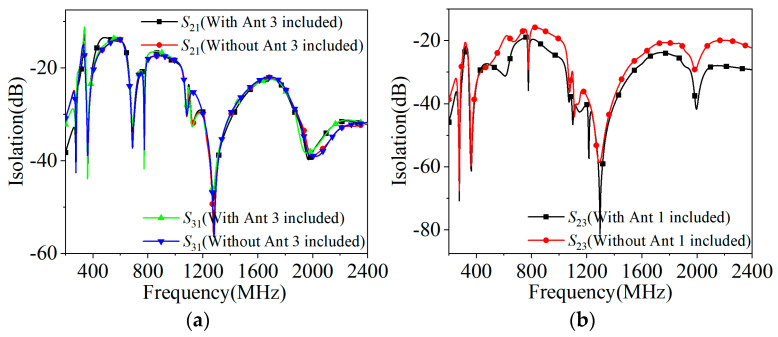
Simulated isolation curves under different port configurations: (**a**) *S*_21_ and *S*_31_ with and without adjacent printed dipoles; (**b**) *S*_23_ with and without the central vertical monopole (Ant 1).

To systematically investigate the mutual coupling within the compact vehicular radome, a rigorous isolation analysis of the three-port MIMO antenna system was conducted (Figure 7).

First, Figure 7a compares the transmission coefficients (*S*_21_ and *S*_31_) with and without the adjacent printed elements. The near-perfect overlap of the curves indicates that Ant 2 and Ant 3 possess high electromagnetic independence, introducing negligible parasitic loading on each other’s coupling paths back to the central monopole (Ant 1).

Second, Figure 7b plots the isolation *S*_23_ between the two parallel printed dipoles. When the central monopole Ant 1 is introduced (black curve), *S*_23_ significantly drops (improving to below −20 dB, with a −80 dB decoupling notch at 1300 MHz). Conversely, when Ant 1 is removed (red curve), the coupling between Port 2 and Port 3 increases by 3–10 dB. This improvement demonstrates that the central vertical metallic monopole effectively acts as a passive electromagnetic shield, blocking the direct line-of-sight near-field coupling between the parallel printed dipoles and dramatically enhancing the isolation of the side MIMO sub-arrays.

## 3. Simulation and Measurement Results

**Figure 8:**
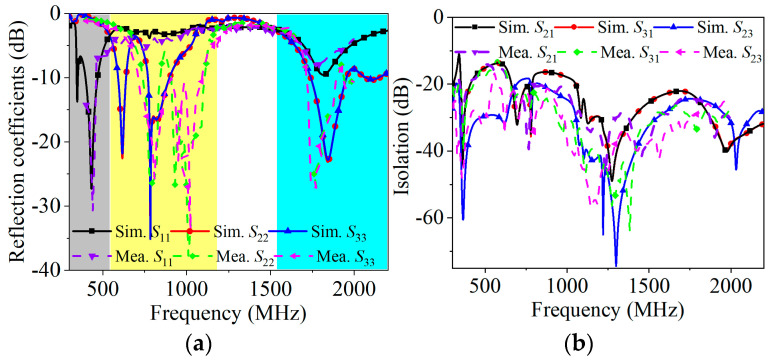
Simulated and measured reflection coefficients and isolation: (**a**) reflection coefficients; (**b**) isolation.

The coefficients were extracted separately for the monopole and the printed elements with all antennas kept in the structure. During measurement, each element was fed individually, and the remaining ports were terminated with 50 Ω matched loads. Figure 8a compares the simulated and measured reflection coefficients of the antenna. The monopole achieves a −10 dB impedance bandwidth of 400–470 MHz. The printed antenna realizes a −10 dB impedance bandwidth of 824.2–879.2 MHz, and its −5.5 dB bandwidth fully covers 1765–1880 MHz. The measured results agree well with the simulations, showing a slight shift due to mutual coupling between antennas and fabrication tolerances.

**Figure 9:**
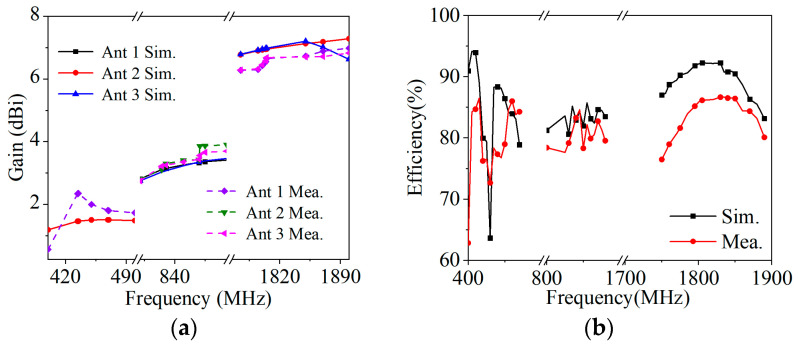
Simulated and measured gain and efficiency of the proposed antenna: (**a**) gain; (**b**) efficiency.

In space-constrained MIMO systems, intense mutual coupling typically dissipates guided power through adjacent ports, degrading the radiation efficiency. In our proposed hybrid architecture, the central monopole excites a vertically polarized omnidirectional mode, whereas the side-printed dipoles generate horizontally dominant tilted polarizations. This spatial and polarization orthogonality minimizes active coupling between ports. As a result, the port isolation remains high, preventing mutual loading and ensuring that the radiation efficiency remains high (>75%) across all operating bands, which is theoretically supported by the polarization diversity. Given the adopted MIMO architecture, inter-element isolation serves as a vital performance index. Satisfactory isolation can be maintained even when the three antennas are deployed in an array within a confined space. As depicted in Figure 8b, the isolation is lower than −10 dB in the 400–470 MHz and 824.2–879.2 MHz bands, and reaches below −20 dB in the high-frequency 1765–1880 MHz band. This level of isolation is highly satisfactory given the spatial constraints imposed by the shark-fin radome. From Figure 9a, it is observed that the measured and simulated gain curves of the proposed antenna exhibit good consistency, and the gain remains favorable throughout the operating frequency range. Figure 9b presents the simulated and measured radiation efficiency of the proposed tri-band antenna. The results show that the radiation efficiency exceeds 75% across the entire UHF band, and stays above 78% and 82% in the LTE Band 5 and LTE-1800 bands, respectively.

**Figure 10:**
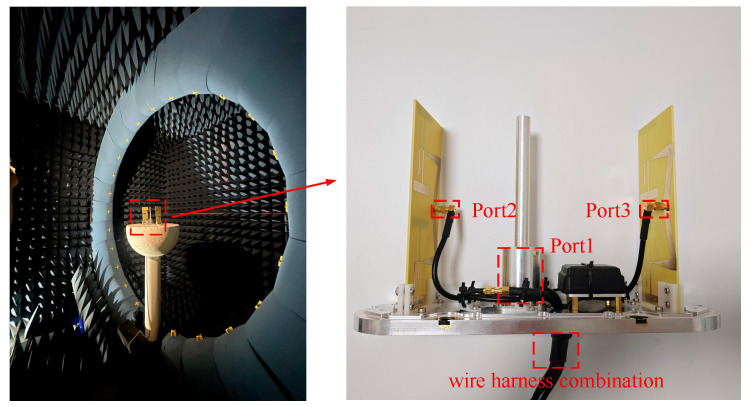
Radiation pattern measurement setup.

**Figure 11:**
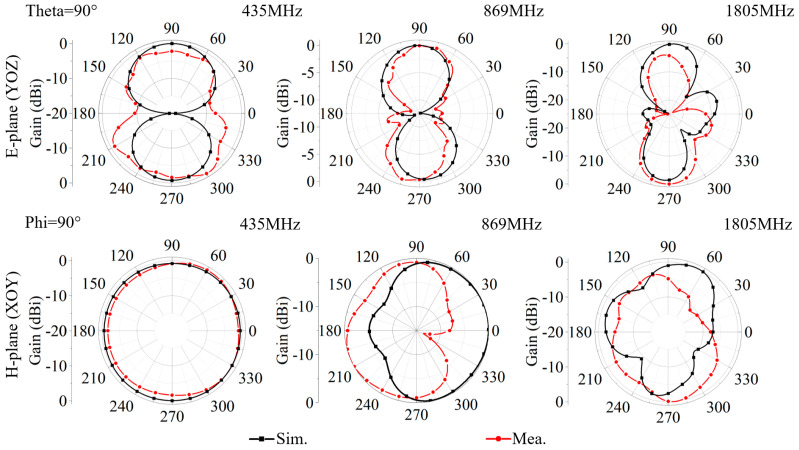
Simulated and measured patterns at different frequencies of the proposed 5G antenna.

To validate the design, a prototype of the proposed antenna is fabricated and measured, as shown in Figure 10. The far-field radiation parameters of the antenna were obtained through microwave anechoic chamber testing. For the manufactured antenna, all feeding ports are assembled with standard 50 Ω straight SMA female coaxial connectors, which are soldered on the antenna’s ground plane to connect test cables. Figure 11 depicts the simulated and measured far-field radiation patterns of the 5G main and diversity antennas in both the E-plane and H-plane at three distinct operating frequencies: 435 MHz, 869 MHz, and 1805 MHz. The simulated results are observed to be in good agreement with the measured far-field radiation patterns, and the minor discrepancies between them can be attributed to manufacturing tolerances and other environmental factors encountered during the experimental measurements.

To further evaluate the performance and demonstrate the advancement of the proposed tri-band shark-fin antenna, a comparative analysis with recently reported vehicular antennas is conducted. Table 2 summarizes the comparison in terms of operating bands, peak gain, physical dimensions, isolation, and omnidirectionality. As shown in the table, while most existing designs focus on the conventional LTE bands, the proposed design successfully integrates the challenging UHF band (400 MHz) within a similar or even more compact volume while maintaining superior gain and port isolation.

## 4. Conclusions

This paper proposes a shark-fin MIMO-LTE-band antenna solution for automotive roof-top applications. The antenna simultaneously covers 400–470 MHz, 824.2–879.2 MHz, and 1765–1880 MHz and is fully integrated inside a shark-fin radome. The system consists of two printed radiators and one monopole, yielding a low-profile, low-cost, and easily manufacturable design. Intended for installation within a shark-fin enclosure mounted on a finite ground plane, the antenna system—without any matching network or dedicated decoupling techniques—demonstrates, through both simulation and measurement, excellent performance in terms of reflection coefficient, radiation pattern, and mutual coupling.

## Figures and Tables

**Table 1:** Geometric parameters of the proposed antenna (unit: mm).

Parameter	Value	Parameter	Value	Parameter	Value
*W*_0_	10	*W*_1_	1	*W*_2_	2.7
*W*_3_	2.1	*W*_4_	9.4	*W*_b_	79.6
*L*_0_	39.6	*L*_1_	35	*L*_2_	31.8
*L*_3_	25.4	*L*_4_	42.8	*L*_5_	131.7
*L*_6_	4.3	*L*_7_	6.6	*L*_b_	190
*R*_1_	3.65	*R*_2_	0.64	*R*_3_	1.03
*R*_4_	8.44	*R*_5_	14.65	*La*	350
*Wa*	100	*Da*	110	*Ha*	200

**Table 2:** Performance comparison between the proposed antenna and reference antennas.

Ref.	Bands (MHz)	Number of Bands	Gain (dBi)	Size (mm^3^)	Isolation (dB)	Omnidirectionality (dB)
[8]	824–960, 1710–2175	Dual-band	0.2–3.6	65 × 32 × 20	N/A	1.8
[14]	617–960, 1710–6000	Dual-band	1.7–5.2	180 × 65 × 49.5	>13	3
[15]	698–2700, 5000–6000	Tri-band	1.5–4.9	120 × 52 × 71	>10	2.3
[18]	617–960, 1700–6000	Dual-band	1.6–5.5	194 × 65 × 70	>14	3.7
[21]	698–960, 1710–2700	Dual-band	1.8–4.5	156 × 78 × 66	>13	1.8
[23]	824–894, 5850–5925	Dual-band	1.6–4.0	156 × 86 × 74	>12	3.1
[26]	617–960, 1710–5000	Dual-band	−1.5–1.8	60 × 39.8 × 15	N/A	1.9
[28]	698–2690	Dual-band	1.5–5.8	155 × 85 × 75	>12	3.5
**This work**	**400–470/824.2–879.2/1756–1880**	**Tri-band**	**2.3–7.2**	**350 × 100 × 200**	**>20**	**2**

## Data Availability

The original contributions presented in the study are included in the article, further inquiries can be directed to the corresponding author.
